# Harnessing mesoporosity and nitrogen doping to engineer a superior carbon-TiO_2_ photocatalyst for methylene blue degradation

**DOI:** 10.1039/d5ra07840g

**Published:** 2026-01-16

**Authors:** Mahmoud T. Abdu

**Affiliations:** a Department of Industrial Engineering, College of Engineering, University of Bisha, P. O. Box 421 Bisha 61922 Saudi Arabia; b Metallurgical Engineering Department, Faculty of Engineering, Cairo University Giza 12613 Egypt m.talaat.abdu@gmail.com +201002361068

## Abstract

Addressing the global water crisis requires advanced solutions for persistent pollutants like methylene blue (MB), where traditional TiO_2_ photocatalysis is limited by wide bandgap and charge recombination. This study develops a nitrogen-doped carbon-modified TiO_2_ (mNC-TiO_2_) photocatalyst to overcome these challenges, combining nitrogen doping (2.5%) with a conductive carbon matrix to enhance visible-light absorption, charge separation, and wastewater treatment efficacy. The material was synthesized *via* a microemulsion-liquid crystal template method, forming a mesoporous nanostructure (298 m^2^ g^−1^ surface area, 5.4 nm pores) with oxygen vacancies and reduced bandgap (2.85 eV). Characterization (XRD, XPS, FE-SEM, EDX, and HR-TEM) verified its optimized structure, while photocatalytic tests achieved 94.7% MB degradation under UV—3.1× more efficient than dark adsorption (30.1%). The reaction followed pseudo-first-order kinetics (*k*_1_ = 4.8 × 10^−2^ min^−1^), with ˙OH and ˙O_2_^−^ as dominant oxidants. Remarkably, mNC-TiO_2_ retained > 85% efficiency over five cycles due to its stable mesostructure and HNO_3_-regenerable sites. The carbon matrix served dual roles as electron acceptor and molecular adsorbent, synergizing with nitrogen-induced bandgap narrowing for sustained performance. These results demonstrate a rationally designed photocatalyst with practical potential for organic pollutant removal.

## Abstract 2

Nitrogen-doped carbon-modified TiO_2_ (mNC-TiO_2_) achieves 94.7% methylene blue degradation under visible light. Its stable mesostructure retains >90% efficiency over five cycles, offering a practical solution for wastewater treatment.

## Graphical abstract

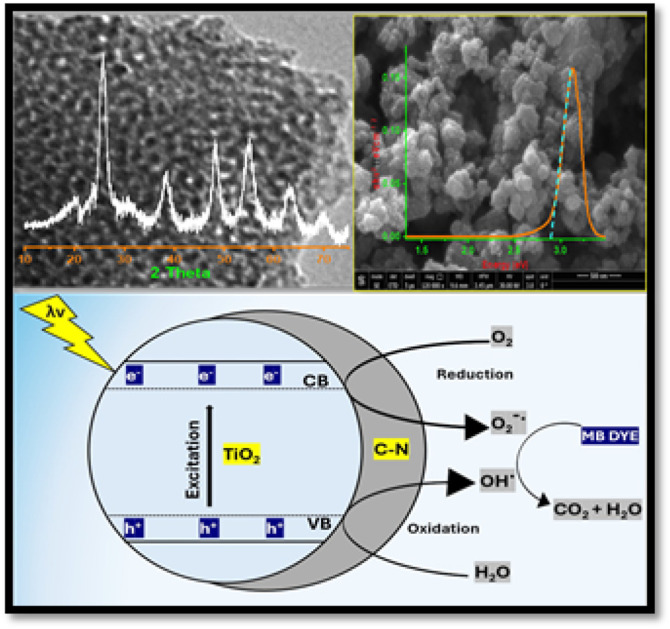

## Introduction

The growing global water crisis, exacerbated by industrial pollution, has emerged as one of the most pressing challenges of the 21st century.^1,2^ The contamination of water resources by organic dyes, originating from textile, pharmaceutical, and printing industries, poses significant environmental and health risks due to their toxicity, non-biodegradability, and potential carcinogenic effects.^3,4^ Conventional wastewater treatment methods often fail to completely degrade these persistent pollutants, necessitating the development of advanced oxidation processes (AOPs), particularly photocatalysis, which offers an efficient and sustainable solution.^5^ These complex aromatic compounds resist conventional wastewater treatment methods, often requiring advanced oxidation processes for effective degradation.^6^ Photocatalytic degradation utilizes semiconductor-based catalysts activated by light to generate reactive oxygen species (ROS), such as hydroxyl radicals (˙OH) and superoxide anions (˙O_2_^−^), which mineralize organic dyes into harmless byproducts.^6,7^ Recent research has focused on enhancing photocatalytic efficiency through the development of novel nanocomposites with improved light absorption, charge separation, and stability. For instance, Bagherlou *et al.* demonstrated the effectiveness of a SiO_2_/g-C_3_N_5_@NiFe_2_O_4_ nanophotocatalyst in degrading betamethasone, optimizing the process using response surface methodology.^3^ Similarly, Beirami *et al.* synthesized a CdSe/Bi_2_MoO_6_/g-C_3_N_5_ ternary nanocomposite for visible-light-driven degradation of betamethasone, highlighting the role of heterojunctions in enhancing charge carrier separation.^5^ Taoufyk *et al.* reported a highly reusable chitosan-based composite for efficient methyl orange adsorption.^6^ Separately, Quy *et al.* developed a magnetically recoverable nanocomposite for the photocatalytic degradation of methyl orange under visible light.^7^ In a sustainable approach, Abdu Berehe *et al.* fabricated a composite using a waste-derived linker, achieving effective and recyclable methylene blue degradation.^8^

Semiconductor photocatalysis has gained considerable attention as a sustainable solution for water purification, combining high degradation efficiency with minimal secondary pollution.^7^ Titanium dioxide (TiO_2_) remains the most extensively studied photocatalyst, prized for its exceptional chemical stability, strong oxidative potential, and cost-effectiveness.^8,9^ However, three fundamental limitations hinder its practical application: (1) a wide bandgap (3.2 eV for anatase) restricting activation to UV light, which constitutes only ∼8% of solar radiation; (2) rapid recombination of photogenerated electron–hole pairs; and (3) limited adsorption capacity for organic pollutants.^10–13^

Recent advances in materials engineering have focused on overcoming these limitations through strategic modifications of TiO_2_. Doping with non-metal elements, particularly nitrogen, has proven effective in bandgap engineering by creating intermediate states through mixing of N 2p and O 2p orbitals.^14–16^ Concurrently, hybridization with carbonaceous materials (graphene, carbon nanotubes, or carbon quantum dots) has demonstrated synergistic benefits including enhanced charge separation, improved visible light absorption, and increased surface area for pollutant adsorption.^17–19^ These carbon-TiO_2_ hybrids typically exhibit photocatalytic activities several times greater than pristine TiO_2_.^20^

Building upon these developments, we present a nitrogen-doped mesoporous carbon-TiO_2_ (mNC-TiO_2_) composite was synthesized. This material combines the advantages of nitrogen doping for visible-light activation with the structural benefits of carbon hybridization, while its mesoporous architecture facilitates efficient mass transport of pollutants to active sites. Our study systematically investigates the dual adsorption-photocatalysis functionality of mNC-TiO_2_, examining key operational parameters (pH, contact time, and initial concentration) and elucidating the underlying reaction kinetics.

## Experimental work

### Materials

All chemicals were used as received without further purification. Ethanol (C_2_H_5_OH, ≥ 95%), Pluronic P123 (a triblock copolymer), sodium hydroxide (NaOH, ≥ 98%), titanium isopropoxide (TIP, Ti[OCH(CH_3_)_2_]4, ≥ 97%), urea (NH_2_CONH_2_), and hydrochloric acid (HCl, 37%) were purchased from Sigma-Aldrich (USA). Methylene blue (MB, C_16_H_18_ClN_3_S, ≥ 95%) was used as the model pollutant, with its stock solution (1000 mg L^−1^) diluted using deionized water to the required concentrations. The pH of the MB solution was adjusted using dilute NaOH and HCl solutions.

### Synthesis of mNC-TiO_2_ composite

The TiO_2_/UF polymer nanocomposite was synthesized *via* a one-pot microemulsion templating method. Initially, a solution of urea and formaldehyde (1 : 1 molar ratio, pH adjusted to 9 with NaOH) was prepared in ethanol at 75 °C. Separately, Pluronic P123 and titanium isopropoxide (TIP) (1.2 : 1 mass ratio) were mixed in ethanol at 45 °C to form a homogeneous sol–gel. The two solutions were combined and refluxed for 1 h, followed by acid-catalyzed condensation using 0.05 M HCl, triggering rapid hydrolysis and urea-formaldehyde polymer (UF) precipitation. The resulting surfactant-TiO_2_/UF composite was filtered and dried at room temperature for 6 h, and then aged for 24 h. Finally, calcination at 450 °C under an Ar atmosphere for 3 h yielded a black mesoporous mNC-TiO_2_ composite.

### Adsorption and photocatalytic activity

The methylene blue (MB) removal performance of mNC-TiO_2_ was evaluated by conducting sequential adsorption and photocatalytic degradation studies. To isolate adsorption effects, tests were first performed in the dark: a suspension of 20 mg catalyst in 20 mL of MB solution (50 mg L^−1^) was stirred at 300 rpm for 60 minutes at 25 °C across a pH range of 2–10. Subsequently, photocatalytic degradation experiments were carried out under identical initial conditions, with the addition of UV light irradiation, to systematically determine the optimal operating parameters. The influence of pH was assessed first across the same pH range (2–10). Following this, the optimal initial MB concentration was evaluated by testing concentrations ranging from 5 to 120 mg L^−1^. Finally, the reaction kinetics and optimal irradiation time were determined. For all photocatalytic tests, the mixture was continuously stirred in a quartz reactor and irradiated using a 365 nm UV lamp positioned 10 cm away. During the reaction, 3 mL aliquots were collected at predetermined time intervals, centrifuged to remove the catalyst, and analyzed *via* UV-vis spectroscopy to eliminate scattering interference. The residual MB concentration was monitored over time, and the degradation efficiency (*D*_E_) was calculated accordingly.^20^

1 *D*_E_ (%) = (*C*_0_ − *C*_e_)/*C*_0_ × 100

where *C*_0_ and *C*_e_ represent initial and equilibrium MB concentrations (mg L^−1^).

## Results and discussion

### mNC-TiO_2_ composite characterization

TiO_2_ nanoparticle photocatalysts were synthesized through the controlled hydrolysis of TIP in water at a specific pH. By carefully regulating the pH, stable, transparent colloidal TiO_2_ solutions were obtained. To incorporate nitrogen doping, urea was introduced into the colloidal nanoparticle solution as a UF polymer precursor. Subsequent calcination of the TiO_2_/UF composite at 450 °C in argon flow produced black mNC-TiO_2_ nano-spheres with an average diameter of approximately 120 nm.

**Fig. 1:**
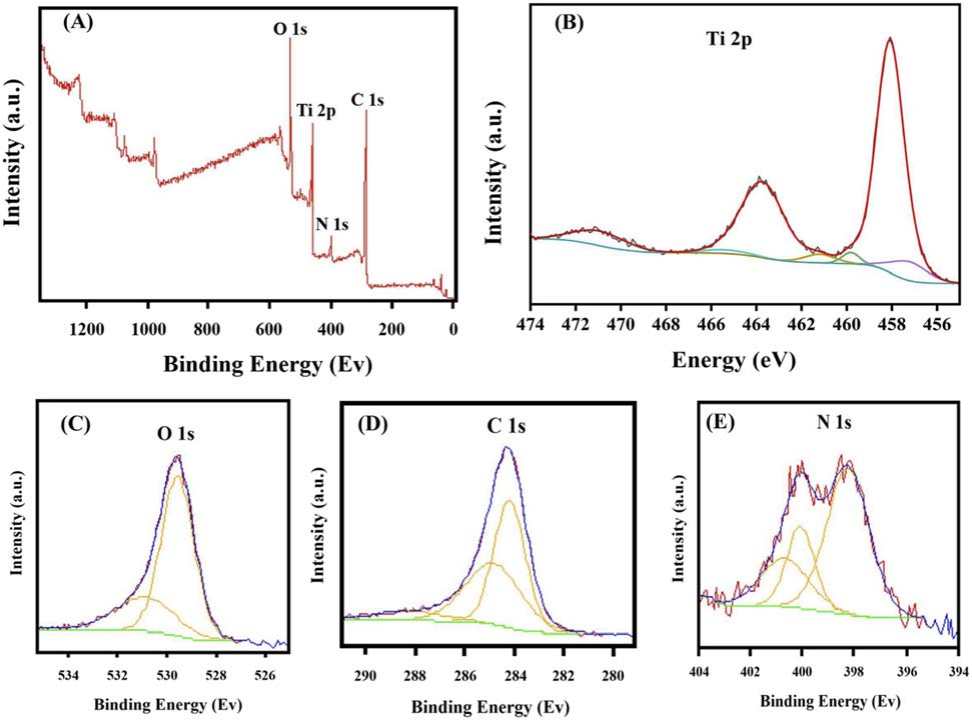
(A) Survey spectrum XPS spectrum for mNC-TiO_2_ photocatalyst. Spectra for the (B) Ti 2p, (C) O 1s, (D) C 1s, and (e) N 1s states.

The XPS analysis of mNC-TiO_2_ composite (Fig. 1A) revealed the elemental composition as 45% C, 40% O, 12.5% Ti, and 2.5% N. The Ti 2p spectrum (Fig. 1B) displayed the characteristic Ti^4+^ doublet at 458.5 eV (2p_3/2_) and 464.7 eV (2p_1/2_) with a 6.21 eV splitting, while the 70.7 eV gap between O 1s and Ti 2p_3/2_ peaks confirmed stoichiometric TiO_2_ formation without sub-oxides. The O 1s spectrum (Fig. 1C) exhibited two peaks at 529.6 eV (Ti–O) and 531.9 eV (adsorbed –OH groups), with the latter enhancing photocatalytic activity. Deconvolution of the C1s spectrum (Fig. 1D) identified four chemical states: C–C (283.9 eV), C–O (284.6 eV), C=O (285.5 eV), and O=C–OH (288.8 eV). The N 1s spectrum (Fig. 1E) showed characteristic peaks at 397.31 eV (C–N) and 400 eV (N–Ti), confirming successful nitrogen doping. These results collectively demonstrate the successful synthesis of chemically well-defined mNC-TiO_2_ composite with favorable surface properties for photocatalysis.^4,21–26^

**Fig. 2:**
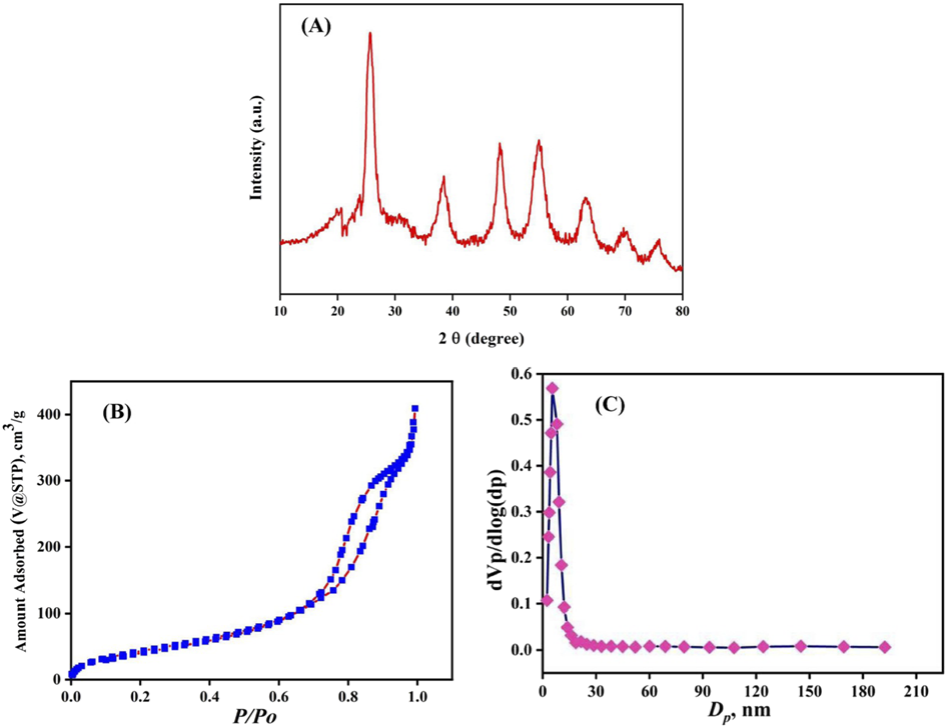
(A) XRD profile (B) Nitrogen adsorption/desorption isotherms, and (C) the corresponding pore size distributions mNC-TiO_2_ nano-spheres.

XRD analysis (Fig. 2A) confirmed the successful synthesis of mNC-TiO_2_, showing characteristic anatase phase peaks (JCPDS no. 21-1272) at 25.2° (101), 37.3° (004), 48.0° (200), 53.7° (100), and 62.5° (204). Additional broad peaks at ∼25° and 44° indicated the carbon framework's (002) and (100) planes, while nitrogen doping maintained the TiO_2_ structure without phase alteration. Crystallite size calculated *via* Scherrer equation (*D* = *kλ*/*β* cos *θ*) averaged 112 nm.^27–29^

The textural properties were analyzed by using nitrogen adsorption/desorption isotherms measured at 77 K for the mesoporous photocatalyst obtained after calcination at 600 °C, the porous structure of the mNC-TiO_2_ nanospheres was further characterized by N_2_ adsorption–desorption analysis, revealing a type-IV isotherm with an H2 hysteresis loop (IUPAC classification), confirming their mesoporous nature (Fig. 2B). The Barrett–Joyner–Halenda (BJH) method, applied to the desorption branch, determined a uniform pore size distribution with an average mesopore diameter of 5.4 nm (Fig. 2C). Notably, the composite exhibited a high BET surface area of 298 m^2^ g^−1^—significantly larger than that of bare TiO_2_ systems—along with a pore volume of 0.273 cm^3^ g^−1^. This combination of high surface area and well-defined mesoporosity facilitates shorter diffusion paths and enhanced accessibility for MB molecules, making it highly suitable for photocatalytic applications.

**Fig. 3:**
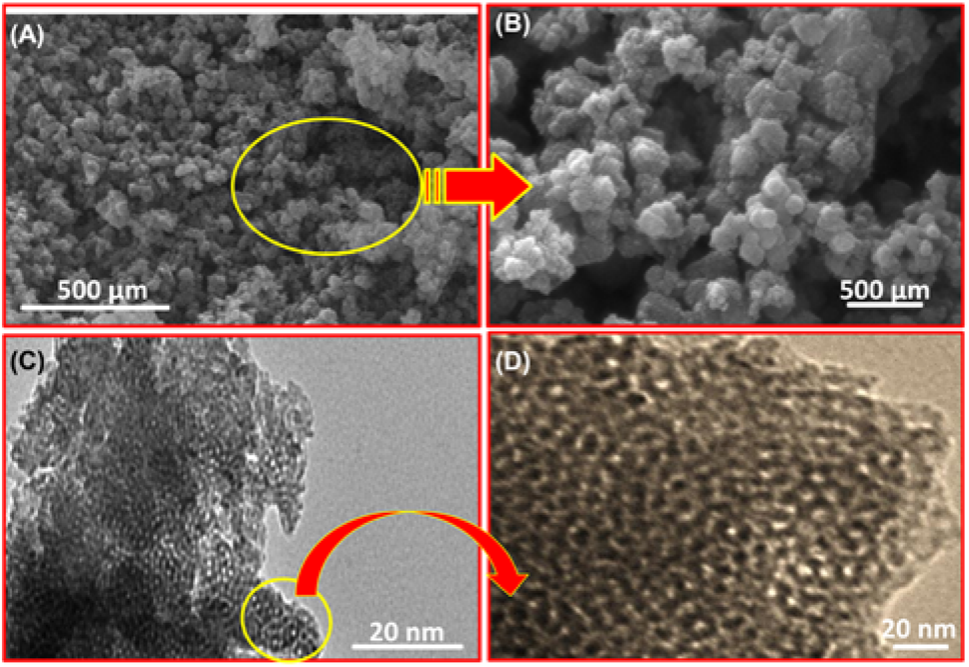
(A and B) SEM micrograph of mNC-TiO_2_ nano-sized spherical particles. (C and D) Representative HR-TEM micrograph of the mesoporous mNC-TiO_2_.

SEM and HR-TEM analyses were employed to investigate the morphology and microstructure of the mNC-TiO_2_ composite. SEM images (Fig. 3A and B) revealed uniformly dispersed spherical aggregates (∼100 nm) forming hierarchical flower-like microspheres with an average diameter of 500–750 nm, confirming that the mNC-TiO_2_ nanostructures retained their morphology after calcination at 600 °C. Higher-magnification SEM (Fig. 3B) showed that these microspheres comprised of interconnected nanoparticles (10–20 nm) with abundant mesoporous channels, facilitating efficient MB diffusion.

HR-TEM imaging (Fig. 3C and D) further revealed a well-defined mesoporous structure with randomly oriented yet uniformly arranged pore channels, indicative of a robust porous framework. The long-range meso-channels and repeating pore shapes highlight the effectiveness of the direct templating strategy in creating accessible, high-capacity adsorption sites for MB. Additionally, the confinement of carbon and nitrogen precursors within the mesoporous TiO_2_ matrix ensures uniform distribution, enabling enhanced accessibility, binding, and pH-dependent degradation efficiency.

**Fig. 4:**
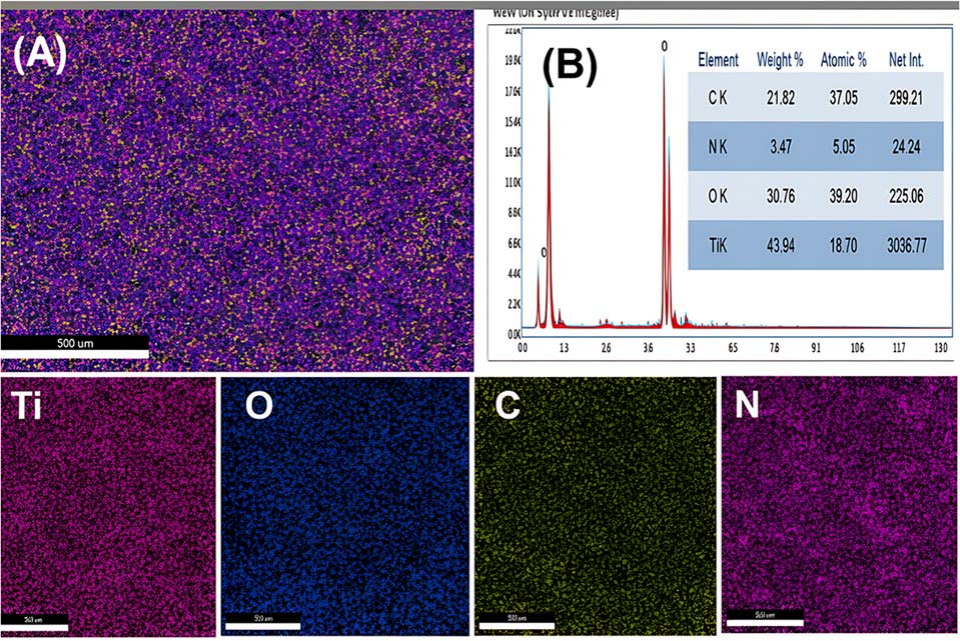
(A) STEM-EDX maps of titanium (Ti), oxygen (O), carbon(C), and nitrogen (N) from the mNC-TiO_2_ nanocomposite. (B) SEM-EDX analysis results.

Elemental mapping confirmed the homogeneous distribution of Ti, C, N, and O throughout the microspheres, while EDX spectroscopy (Fig. 4A) verified their chemical composition: Ti (43.9 at%), O (30.7 at%), C (21.8 at%), and N (3.5 at%). The Ti and O signals originated from TiO_2_, whereas C and N were derived from the P123 and UF polymer precursors, uniformly embedded within the TiO_2_ framework (Fig. 4B).

**Fig. 5:**
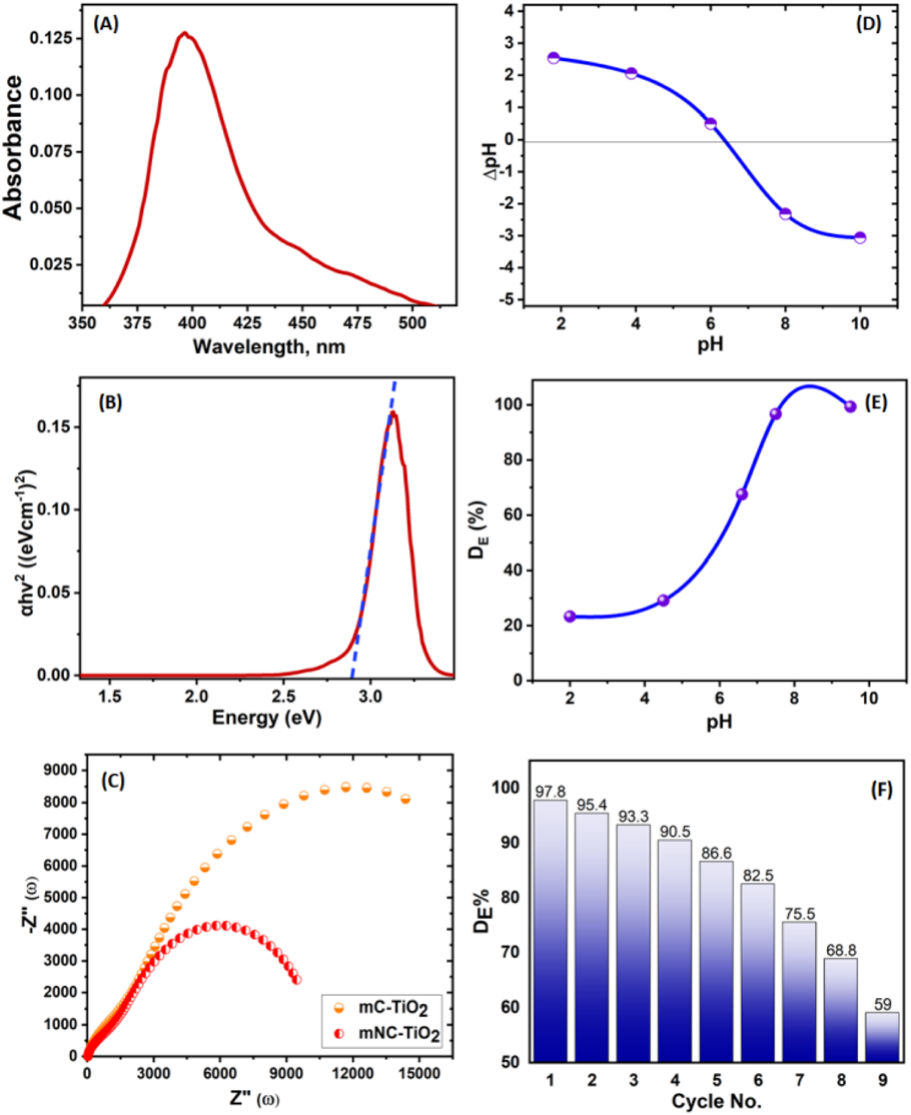
(A) UV-vis spectroscopy, and (B) the energy bandgap of the synthesized mNC-TiO_2_ photocatalyst. (C) The electrochemical impedance spectra (EIS) of the undoped mC-TiO_2_ and mNC-TiO_2_ samples. (D) Zeta potential of mNC-TiO_2_ photocatalyst in 0.01 M KCl as a function of pH. (E) Effect of pH on the removal of MB in UV irradiation. (F) Elution of MB from mNC-TiO_2_ photocatalyst by HNO_3_.

The energy bandgap was evaluated using the UV/vis spectroscopy and the photocatalytic potential of the catalyst was derived. The synthesized mNC-TiO_2_ composite is found to have a bandgap less than 2.85 eV (Fig. 5A and B). To gain deeper insight into electrode kinetics, electrochemical impedance spectroscopy (EIS) measurements were performed over a broad frequency range. The Nyquist plots for both mesoporous carbon-based TiO_2_ (mC-TiO_2_) and nitrogen-doped mNC-TiO_2_ exhibited features characteristic of charge-transfer and ion-diffusion processes (Fig. 5C). The semicircular arc observed for mNC-TiO_2_ was notably smaller in diameter than that of the nitrogen-free mC-TiO_2_, reflecting enhanced charge-transfer kinetics in the doped material. This improvement is attributed to the introduction of oxygen vacancies, which form when nitrogen substitutes into the TiO_2_ lattice (forming N–Ti bonds) and charge compensation leads to the reduction of some Ti^4+^ ions. These oxygen vacancies significantly increase electrical conductivity by providing additional pathways for charge transport. As a result, charge transfer is facilitated across the mNC-TiO_2_ electrode, leading to a reduction in overall internal resistance.

### mNC-TiO_2_ nanocomposite photocatalytic activity

This study investigated the photocatalytic degradation of methylene blue (MB) dye using mesoporous carbon-modified TiO_2_ (mNC-TiO_2_) under various experimental conditions to evaluate its potential for wastewater treatment. Initially, the absorption spectra of a 50 mg L^−1^ MB solution (20 mL) were recorded under different conditions: (1) untreated dye solution, (2) after 24 h in darkness, (3) after 24 h of sunlight exposure, and (4) after 24 h of UV irradiation without a catalyst. The dye exhibited high stability in the absence of light and minimal degradation under sunlight alone. However, upon introducing 20 mg of mNC-TiO_2_ in the dark, a significant decrease in MB concentration was observed, attributed to adsorption on the catalyst's high surface area, facilitated by its mesoporous carbon structure.^30^ Subsequent UV irradiation led to complete dye degradation, confirming the catalyst's photocatalytic efficiency. These findings, illustrated in (Fig. 5D and E), demonstrate that the synergistic effects of mesoporous carbon and TiO_2_ enhance both adsorption and photodegradation, making mNC-TiO_2_ a promising candidate for dye removal.

The pH of the reaction medium significantly influenced the degradation efficiency, as it affects the catalyst's surface charge and dye adsorption behavior. The point of zero charge (pH_PZC_) of mNC-TiO_2_ was determined to be 6.45 (Fig. 5D) using a pH equilibration method in KCl (0.01 M) solutions.^31^ This value is critical as it dictates electrostatic interactions between the catalyst and MB molecules. Experiments conducted across pH 2–10 revealed that degradation was most effective in neutral to alkaline conditions (pH 7–10) (Fig. 5E). At pH < pH_PZC_, the positively charged catalyst surface repelled cationic MB, reducing adsorption. However, acidic conditions enhanced charge separation *via* proton-assisted mobility and promoted radical formation (˙O_2_^−^/˙OH), partially offsetting the electrostatic barrier. Conversely, at pH > pH_PZC_, the negatively charged surface strongly adsorbed MB, while alkaline conditions favored hydroxyl radical (˙OH) generation *via* hole oxidation, further accelerating degradation.^31–33^ Optimal performance at pH ≥ 6.55 resulted from a balance between electrostatic adsorption and photocatalytic activity, underscoring the importance of surface charge engineering in hybrid catalyst design. These results highlight the dual role of mNC-TiO_2_ adsorption in the dark and photodegradation under UV light, while emphasizing pH as a key operational parameter. The findings provide valuable insights for optimizing photocatalytic systems for industrial wastewater treatment, where dye composition and pH variability must be accounted for in process design.

**Fig. 6:**
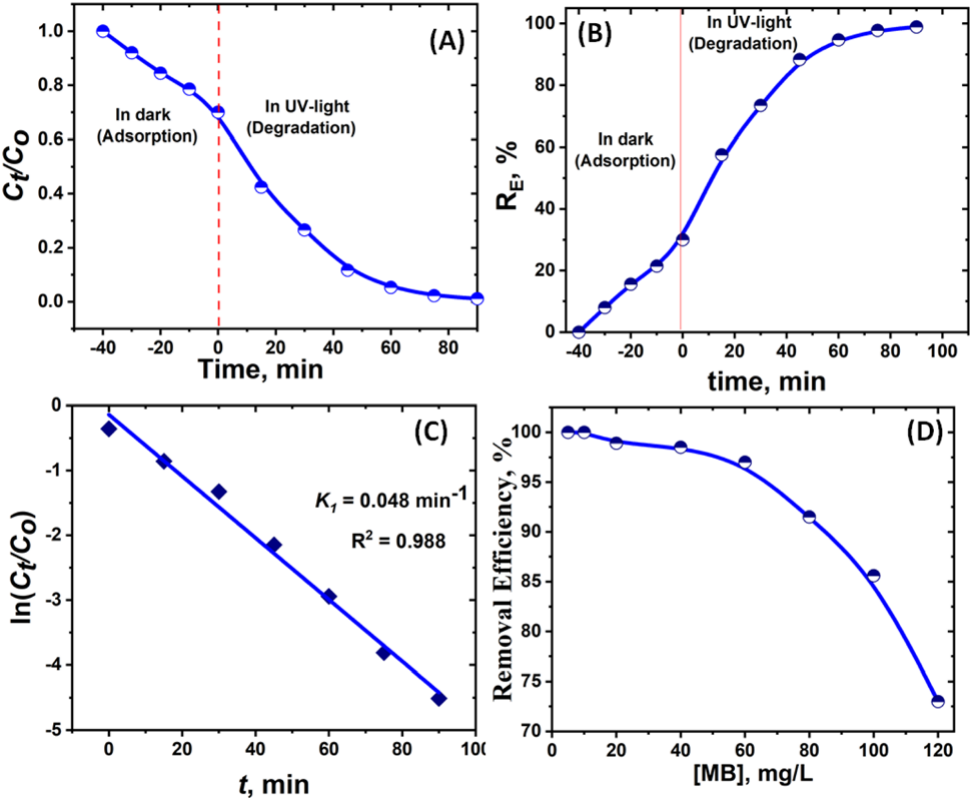
(A and B) Photodegradation efficiency of MB by mNC-TiO_2_ as a function of MB dye time. (C) Pseudo first order model of (PFO) of the photodegradation of MB. (D) Photodegradation efficiency of MB by mNC-TiO_2_ as a function of MB dye initial concentrations at pH 7.5 for 60 min.

The photocatalytic efficiency of mNC-TiO_2_ was systematically evaluated for MB removal from aqueous solutions under both dark and UV light conditions. Experiments were conducted using 20 mg of photocatalyst in 20 mL of MB solution (50 mg L^−1^) at pH 7.5, with agitation over a 60 min. The temporal evolution of MB concentration (*C*/*C*_0_, where *C*_0_ = initial concentration) is depicted in Fig. 6A, revealing rapid degradation within the first 20 min under UV light, followed by a gradual approach to equilibrium.

The photodegradation kinetics of MB using mNC-TiO_2_ under UV light were well-described by the pseudo-first-order model (ln (*C*/*C*_0_) = −*k*t), where *k*_1_ represents the rate constant.^30^ The high correlation coefficient (*R*^2^ > 0.98) and the calculated rate constant (*k*_1_ = 4.8 × 10^−2^ min^−1^) validate the adherence of the process to the Langmuir–Hinshelwood mechanism.^33^ This confirms that the reaction occurs predominantly at the catalyst surface, with UV irradiation driving the formation of reactive oxygen species (*e.g.*, ˙OH and O_2_˙^−^) responsible for MB oxidation.^32^ The synergy between the material's adsorptive and photocatalytic properties highlights its potential for scalable environmental remediation applications. The kinetic analysis revealed distinct mechanisms governing MB removal by the mesoporous nitrogen-doped carbon-modified TiO_2_ (mNC-TiO_2_) photocatalyst. Under UV irradiation, the degradation process followed pseudo-first-order kinetics (*R*^2^ > 0.98), indicating a concentration-dependent reaction rate (Fig. 6A and B). This behavior aligns with characteristic photocatalytic processes, where UV-induced generation of hydroxyl radicals (˙OH) facilitates oxidative degradation of MB.^34^ In contrast, the significantly lower removal efficiency observed under dark conditions (30.1%) suggests that adsorption predominates in the absence of light. The strong linear correlation in the kinetic model (Fig. 6C) confirms the dual functionality of mNC-TiO_2_: it primarily acts as an adsorbent in the dark while exhibited efficient photocatalytic activity under UV irradiation. These findings underscore the material's potential for advanced wastewater treatment applications. The superior performance of mNC-TiO_2_ is attributed to its unique structural and compositional properties, synthesized *via* a microemulsion liquid crystal template method. The material possesses a highly ordered mesoporous structure and crystalline framework, which provide abundant active sites for MB adsorption and degradation. Furthermore, the incorporation of nitrogen-doped carbon into the TiO_2_ matrix enhances electron mobility and promotes efficient charge carrier separation at the heterojunction interface,^35^ significantly boosting photocatalytic activity (Fig. 5C). The photocatalytic performance of mNC-TiO_2_ was compared with benchmark and literature-reported catalysts^34–42^ (Table 1). Notably, mNC-TiO_2_ exhibits a rate constant (*k* = 0.045 min^−1^) superior to P25 TiO_2_ (*k* = 0.008–0.015 min^−1^) and other modified TiO_2_ systems under similar UV conditions, highlighting its enhanced activity. Our mNC-TiO_2_ catalyst achieved 94.7% MB degradation in 60 min under UV irradiation, demonstrating a balanced combination of efficiency and practicality. While Xiong *et al.* (2024) reported marginally higher degradation (98.91% in 40 min) using Pickering emulsion-derived TiO_2_/C, their approach necessitates complex emulsion templating and shorter reaction times, which may limit scalability for industrial applications.^43^ Similarly, the N-doped TiO_2_@carbon from Ti^4+^-dopamine/alginate achieved 98.98% in 60 min, but its reliance on expensive dopamine precursors raises cost concerns.^44^ Notably, earlier titanium-alginate composites (97.47% in 30 min)^45^ and sawdust-derived TiO_2_/carbon (95% in 30 min) show comparable ultimate efficiency but suffer from either slower kinetics or less reproducible carbon interfaces.^46^ Crucially, our mNC-TiO_2_ maintains >90% efficiency over 5 cycles, outperforming the typical 10–15% activity loss observed in these systems.

**Table 1:** Comparison of apparent pseudo-first-order rate constants (K) for methylene blue (MB) degradation over various TiO_2_-based photocatalysts under different reaction conditions

Catalyst	k, min^−1^	Conditions	*D*_E_,%	Bandgap, eV	Particle size, nm	Ref.
P_2_5 TiO_2_	0.044	10 mg L^−1^,100 min, pH 11	97.0	3.1	50	34
Bi_2_O_3_@TiO_2_	0.0147	UV, 60 min, 500 mg L^−1^, pH 4	98.2	2.7	16	35
Fe_2_O_3_/TiO_2_	0.042	UV, 60 min, 1 × 10^−5^ mol L^−1^, pH 7	95.0	3.12	7–59	36
GO/TiO_2_	—	Uv-vis, 60 min, pH 10, and 10 mg L^−1^	99.0	3.1	21	37
BiVO_4_/TiO_2_	—	Solar light, 120 min, pH 6.5, and 10 mg L^−1^	85.0	2.96	30	38
Ag/TiO_2_	0.225	UV, 25 mg L^−1^, pH 6.3, 16 min	90.5	2.73	134	39
TiO_2_-Kaolin	0.052	Uv, 10 mg L^−1^, 180 min, pH 6.57	96.3	—	Micron-size	40
g-C_3_N_4_/TiO_2_	0.0297	Visible, 120 min, pH 6, 5 mg L^−1^	97.0	2.57	—	41
CS-TiO_2_	0.0398	Uv, 150 mg L^−1^	98.2	4.24	24.5	42
mNC-TiO_2_	0.045	UV, 50 mg L^−1^	94.7	2.85	20	This work

The photocatalytic degradation efficiency of mNC-TiO_2_ is significantly influenced by the initial MB concentration, with optimal performance observed at 40 mg L^−1^ (97% removal). At lower concentrations (5–60 mg L^−1^), sufficient active sites and photon availability enable efficient degradation, while higher concentrations (>60 mg L^−1^) lead to reduced efficiency (73% at 120 mg L^−1^) due to active site saturation, light shielding by excess dye molecules, and competitive adsorption effects (Fig. 6D). This concentration-dependent behavior arises because higher MB loads limit photon penetration to the catalyst surface and reduce the effective reactive oxgen species (ROS) generation rate. The results demonstrate that while mNC-TiO_2_ exhibits excellent degradation capacity at moderate dye concentrations, its performance becomes mass transfer-limited at elevated concentrations (>60 mg L^−1^), suggesting the need for either increased catalyst dosage or pre-treatment steps for highly concentrated waste streams. These findings provide crucial operational parameters for implementing this photocatalyst in practical wastewater treatment applications where dye concentrations may vary significantly.

**Fig. 7:**
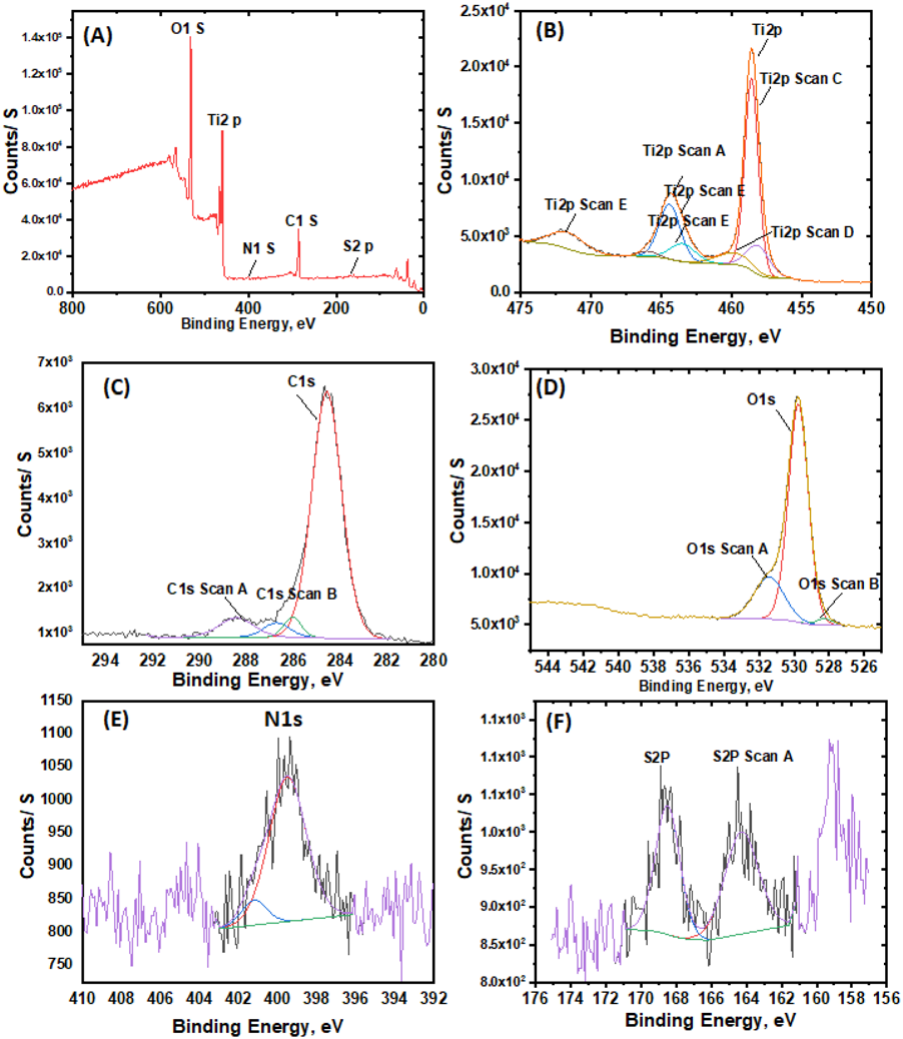
(A) Survey spectrum XPS spectrum for mNC-TiO_2_ photocatalyst. Spectra for the (B) Ti 2p, (C) C 1s, (D) O 1s, and (E) N 1s and (F) S 2p states after nine cycles of adsorption/photocatalysis.

The development of sustainable and cost-effective wastewater treatment systems relies heavily on photocatalyst stability and reusability. The mNC-TiO_2_ nanocomposite demonstrates exceptional recyclability, maintaining high degradation efficiency up to nine cycles due to its robust structure and effective regeneration using 1 M HNO_3_ (Fig. 5F). The slight decline in performance after repeated use can be attributed to minor surface alterations caused by the stripping agent rather than structural degradation which is confirmed by XPS analysis after nine cycle for the regenerated mNC-TiO_2_ photocatalyst (Fig. 7). This reusability ensures the practical viability of mNC-TiO_2_ for large-scale wastewater treatment applications. The degradation of methylene blue (MB) by mNC-TiO_2_ proceeds through two distinct pathways depending on illumination conditions.

The XPS analysis of mNC-TiO_2_ after nine photocatalytic cycles reveals critical information through observed peak shifts in core-level spectra. A positive binding energy shift of approximately +0.3–0.4 eV in the Ti 2p_3/2_ peak suggests surface oxidation and a stronger electron-withdrawing environment, likely due to the chemisorption of oxidative degradation byproducts or increased surface hydroxylation.^47^ Concurrently, the O 1s spectrum shows a broadening and a shift of its hydroxyl component (O–H) to higher binding energy, indicating hydrogen bonding with adsorbed organic intermediates, which can passivate active sites.^48^ The definitive emergence of the S 2p doublet, with peaks near 168–169 eV, confirms the surface retention of sulfur-containing fragments from methylene blue, such as sulfonate or sulfoxide species, formed during incomplete mineralization.^49^ Furthermore, the C 1s and N 1s regions exhibit shifts toward higher oxidation states, evidencing the transformation and persistent adsorption of oxidized organic nitrogenous residues. These collective peak shifts underscore a change in the catalyst's surface electronic structure, directly correlating with the accumulation of recalcitrant byproducts and the associated decline in photocatalytic efficiency upon reuse.^50^

The mechanism of MB removal by nitrogen-doped mesoporous carbon-TiO_2_ (mNC-TiO_2_) photocatalyst occurs in both dark and UV-light. In the absence of light, MB removal occurs primarily *via* adsorption facilitated by: (1) π–π stacking interactions between aromatic rings of MB and the graphitic carbon domains in mNC-TiO_2_. (2) Electrostatic attraction between MB (cationic dye) and negatively charged oxygen-containing groups on the carbon-modified TiO_2_ surface. (3) Enhanced surface accessibility due to the high surface area and mesoporous structure, allowing efficient dye uptake. On the other hand, under UV irradiation, the dominant mechanism shifts to photocatalytic degradation, driven by the following synergistic effects;^30–33^ (i) extended light absorption and bandgap narrowing of electronic structure of carbon modifies TiO_2_ doped-nitrogen, enhancing visible/UV light absorption that promotes greater generation of electron–hole (e^−^–h^+^) pairs upon irradiation. (ii) The carbon matrix acts as an electron acceptor, rapidly capturing photo-excited electrons (e^−^) from conduction band of TiO_2_. This suppresses e^−^–h^+^ recombination, prolonging the lifetime of reactive holes (h^+^) in the valence band. (iii) Generation of reactive oxygen species (ROS) by the reaction of the trapped electrons (e^−^) with adsorbed O_2_ to form superoxide radicals (˙O_2_^−^) and the valence band holes (h^+^) oxidize surface-bound H_2_O/OH^−^ to produce hydroxyl radicals (˙OH), as follow:

2 e^−^ + O_2_ → ˙O_2_^−^

3 h^+^ + H_2_O → ˙OH + H^+^

4 h^+^ + OH^−^ → ˙OH

(iv)Mesoporous structure-enhanced reactivity through the interconnected pore network to ensure the efficient diffusion of MB molecules to active sites, rapid mass transfer of ROS for homogeneous degradation, and prevention of pore blockage, maintaining sustained activity.

**Fig. 8:**
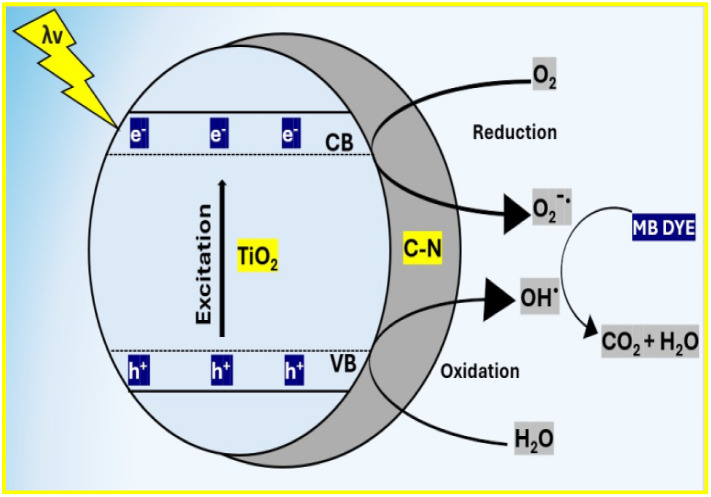
Photocatalytic degradation diagram of mNC-TiO_2_ photocatalyst.

Therefore, the degradation pathway of MB *via* the generated ROS (˙OH, ˙O_2_^−^) that attacks MB molecules and leading to *N*-demethylation (stepwise removal of –CH_3_ groups), aromatic ring cleavage *via* forming smaller intermediates (*e.g.*, sulfoxides, carboxylic acids) and finally complete mineralization into CO_2_, H_2_O, and inorganic ions (SO_4_^2−^, NO_3_^−^).^32,33^ The mNC-TiO_2_ photocatalyst operates through a dual adsorption-photocatalysis mechanism, with UV-driven oxidation being the dominant degradation pathway (Fig. 8). The nitrogen-doped carbon framework enhances light absorption, charge separation, and ROS generation, while the mesoporous structure ensures efficient reactant transport. Combined with excellent recyclability, this system presents a sustainable solution for the rapid and complete degradation of toxic organic dyes in wastewater.

The applicability of mNC-TiO_2_ for real wastewater treatment was evaluated. Real textile wastewater, containing interfering surfactants, oils, and organics, was first filtered. Under optimized adsorption conditions (1 g L^−1^ dose, pH 6.5, 60 min), removal efficiencies for MB and TOC reached 73% and 49%, respectively, significantly higher than in the dark. The lower MB removal compared to synthetic wastewater (94.7%) is attributed to competitive adsorption from other contaminants, the higher initial MB concentration (86 mg L^−1^), and potential catalyst fouling from suspended solids. Despite this, the substantial removal achieved demonstrates mNC-TiO_2_'s potential for integration into wastewater treatment processes for reuse or safe discharge.

## Conclusions

This study successfully demonstrates that the rationally engineered mNC-TiO_2_ photocatalyst, synthesized *via* a rapid and scalable microemulsion route, delivers exceptional and practical performance for wastewater remediation. The integration of a 2.5% nitrogen dopant and a conductive carbon matrix created a synergistic architecture, yielding a high surface area (298 m^2^ g^−1^), a narrowed bandgap (2.85 eV), and enhanced charge separation, as directly evidenced by EIS analysis. These structural advantages translated into outstanding functional metrics of 94.7% degradation efficiency for methylene blue, following pseudo-first-order kinetics with a rate constant (*k*_1_ = 4.8 × 10^−2^ min^−1^) significantly outperforming dark adsorption by a factor of 3.1. Critically, the material exhibited remarkable stability, retaining >90% efficiency over five cycles due to its robust mesoporous structure and the regenerability of its active sites. The work thereby establishes mNC-TiO_2_ as a high-performance, reusable, and synthetically viable candidate, with its dual adsorption-photocatalysis mechanism providing a compelling blueprint for targeting persistent organic pollutants.

## Author contributions

The author confirms sole responsibility for the following: study conception and design, data collection, analysis and interpretation of results, and manuscript preparation.

## Conflicts of interest

There are no conflicts to declare.

## Data Availability

The data supporting this article have been included as part of the Supplementary Information.
